# Cyclin Dependent Kinase-1 (CDK-1) Inhibition as a Novel Therapeutic Strategy against Pancreatic Ductal Adenocarcinoma (PDAC)

**DOI:** 10.3390/cancers13174389

**Published:** 2021-08-30

**Authors:** Rosa Wijnen, Camilla Pecoraro, Daniela Carbone, Hamid Fiuji, Amir Avan, Godefridus J. Peters, Elisa Giovannetti, Patrizia Diana

**Affiliations:** 1 Department of Medical Oncology, Cancer Center Amsterdam, Amsterdam UMC, VU University Medical Center (VUmc), 1081 HV Amsterdam, The Netherlands; rosavan2000@gmail.com (R.W.); camilla.pecoraro@unipa.it (C.P.); gj.peters@amsterdamumc.nl (G.J.P.); 2 Dipartimento di Scienze e Tecnologie Biologiche Chimiche e Farmaceutiche (STEBICEF), Università degli Studi di Palermo, 90123 Palermo, Italy; daniela.carbone@unipa.it; 3 Department of Biochemistry, Payame-Noor University, Mashhad 19395-4697, Iran; hamid_fiuji@yahoo.com; 4 Metabolic Syndrome Research Center, Mashhad University of Medical Science, Mashhad 91886-17871, Iran; AvanA@mums.ac.ir; 5 Department of Biochemistry, Medical University of Gdansk, 80-210 Gdansk, Poland; 6 Cancer Pharmacology Lab, AIRC Start Up Unit, Fondazione Pisana per la Scienza, 56124 Pisa, Italy

**Keywords:** pancreatic cancer, PDAC, CDK1 inhibition, cell cycle regulation, novel treatment

## Abstract

**Simple Summary:**

Pancreatic ductal adenocarcinoma (PDAC) is one of the most lethal cancers in humans, due to late diagnosis and limited treatment possibilities. Improved treatment for PDAC patients is warranted. Cyclin-dependent kinase 1 (CDK1) is a stimulator of cell cycle progression and its activity is regularly enhanced in pancreatic cancer cells. Therefore, CDK1 has been proposed as a novel drug target to treat patients with PDAC. This review describes the potential of CDK1 inhibition as a treatment for PDAC by outlining the molecular pathways influenced by CDK1 inhibition and new therapeutic strategies.

**Abstract:**

The role of CDK1 in PDAC onset and development is two-fold. Firstly, since CDK1 activity regulates the G2/M cell cycle checkpoint, overexpression of CDK1 can lead to progression into mitosis even in cells with DNA damage, a potentially tumorigenic process. Secondly, CDK1 overexpression leads to the stimulation of a range of proteins that induce stem cell properties, which can contribute to the development of cancer stem cells (CSCs). CSCs promote tumor-initiation and metastasis and play a crucial role in the development of PDAC. Targeting CDK1 showed promising results for PDAC treatment in different preclinical models, where CDK1 inhibition induced cell cycle arrest in the G2/M phase and led to induction of apoptosis. Next to this, PDAC CSCs are uniquely sensitive to CDK1 inhibition. In addition, targeting of CDK1 has shown potential for combination therapy with both ionizing radiation treatment and conventional chemotherapy, through sensitizing tumor cells and reducing resistance to these treatments. To conclude, CDK1 inhibition induces G2/M cell cycle arrest, stimulates apoptosis, and specifically targets CSCs, which makes it a promising treatment for PDAC. Screening of patients for CDK1 overexpression and further research into combination treatments is essential for optimizing this novel targeted therapy.

## 1. Introduction

With a 5-year survival rate of only 10 percent, pancreatic cancer is one of the most lethal cancers in humans [1]. The American Cancer Society estimates that in the United States alone, 48,220 people will die as a result of pancreatic cancer in 2021 [2]. Currently pancreatic cancer is the seventh leading cause of global cancer deaths in both sexes, and is the most lethal of common malignancies, with incidence and mortality rates nearly equivalent [3]. Unlike most other forms of cancer, pancreatic cancer has seen only minor improvement in survival rates over the past 40 years [4]. These poor survival rates are largely attributable to a late diagnosis of the disease and limited treatment possibilities. Surgery represents the only curative treatment, but only 20 percent of pancreatic tumors are eligible for resection [5,6]. The current most commonly-used treatment options—FOLFIRINOX (folinic acid, fluorouracil, irinotecan, and oxaliplatin) and gemcitabine plus nab-paclitaxel—are highly toxic and not effective enough [7,8]. The detrimental impact of pancreatic cancer combined with the low availability of treatment options has caused the urgent need to develop novel therapeutic strategies. 

Pancreatic ductal adenocarcinoma (PDAC) develops from differentiated pancreatic ductal cells and accounts for 90 percent of all pancreatic tumors. The disease advances in multiple stages, which are characterized by histopathological and molecular changes in the pancreatic duct cell lining. Through these stages, healthy epithelium transforms first into pancreatic intraepithelial neoplasia, followed by invasive carcinoma [9]. 

**Figure 1:**
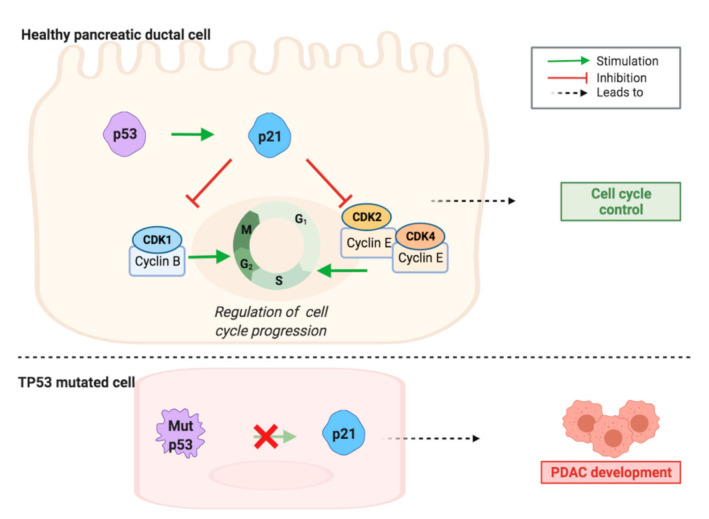
TP53 gene mutation contributes to loss of cell cycle regulation and PDAC formation and development. A healthy cell expresses normal p53, which influences cell cycle control via stimulation of p21, which can inhibit CDK/cyclin complexes. The CDK1/cyclin B complex promotes G2/M phase progression, whereas CDK2/cyclin E and CDK4/cyclin E complexes promote G1/S phase progression. Via loss of CDK/cyclin complexes inhibition, mutated p53 contributes to loss of cell cycle control, which can lead to PDAC development.

In 78% of the PDAC cases, mutations linked to cell cycle regulation occur [10]. Of these, tumor suppressor protein p53 (TP53) is one of the most frequent and relevant drivers of pancreatic tumorigenesis, as it is mutated in 70% of the PDAC cases. TP53 is a tumor suppressor gene that codes for p53, a molecule involved in cell cycle regulation [11]. Of note, loss of the TP53 appears to occur late (after KRAS and CDKN2A alterations) in the classical model of development of pancreatic neoplasia [7]. However, loss of heterozygosity at chromosome 17p (the location of the TP53 gene) as well as abnormalities of TP53 gene expression have all also been reported in pancreatic duct lesions, and a recent model suggests that PDAC progression is neither gradual nor follows the accepted mutation order because most tumors harbor complex rearrangement patterns associated with mitotic errors [8]. One of its downstream effects is cyclin-dependent kinase 1 (CDK1) inhibition, via p21 activation. This effect is lost when the TP53 gene is mutated, mutations fail to stimulate p21, thus CDK1 is no longer inhibited (Figure 1). CDK1 stimulates progression through the cell cycle [12]. When stress or DNA damage occurs, TP53 blocks CDK1 signaling, thus ultimately causing apoptosis. These properties of CDK1 suggest that in the opposite scenario, overexpression of the protein could result in replication of cells with faulty DNA, causing cancer cell proliferation. Indeed, scientists have identified sustained CDK1 activity as essential for tumorigenesis [13].

CDK1 genes are significantly overexpressed in tumor cells of PDAC patients [14], which is associated with more advanced stages of PDAC and is an indicator of poor survival rates for patients. Moreover, inhibition of CDC25, an activator of CDK1, leads to a reduction in growth of pancreatic cancer cell lines [15]. Together, these studies indicate the potential of CDK1 inhibition as a novel drug target to treat PDAC. In addition, several other studies indicate the potential of CDK1 inhibition for both cancer treatment in general [16,17], and specifically for PDAC [18,19]. 

However, there is only scarce clinical evidence for the concrete benefits of CDK1 inhibition [13]. Cytotoxic effects of CDK1 inhibitors on healthy cells indicate that the safety of CDK1 inhibition should be investigated [20]. Furthermore, there is limited research that thoroughly explains the molecular mechanisms through which CDK1 overexpression induces PDAC development and how CDK1 inhibition could combat this. Additionally, different PDAC subtypes may have different behaviors and targetability, but there are few studies focusing on these subtypes [21]. These questions should be explored further in order to reach a consensus on whether CDK1 inhibition is a good drug target to treat patients with PDAC, which is lacking in the existing literature. This literature review aims to assess the potential of CDK1 inhibition by outlining how enhanced activity of this kinase contributes to the development and progression of PDAC, and how a novel CDK1 inhibitor would influence the molecular pathways involved in PDAC to exert its proposed anticancer mechanism. 

## 2. Tumorigenic Activity of CDK1 and Anticancer Mechanisms of CDK1 Inhibitors

### 2.1. CDK1 and Cell Cycle

Cyclin-dependent kinases (CDKs) are serine/threonine kinases which play a crucial role in regulating the cell cycle [12]. They depend on cyclins, separate protein subunits with which the kinases form CDK/cyclin complexes [22]. These complexes can control the cell cycle process by either stimulating or halting cell cycle progression. CDK activity is regulated by several factors, including the availability of their cyclin partners, the presence of inhibitory tyrosine phosphorylation, and the activity of CDK inhibitors [23]. There are currently more than 20 identified family members of CDKs, which share the catalytic domain formed by an ATP-binding pocket, the cyclin-binding domain, and the activation loop called T-loop motif [22]. CDK/cyclin complex formation induces a conformational change of the T-loop, which leads to the exposure and phosphorylation of the substrate-binding domain of the kinase. CDKs, besides the cell cycle, also influence other cellular and developmental processes [12]. These processes include stem cell self-renewal, transcription, epigenetic regulation, neuronal functions, and spermatogenesis. 

CDK1—also known under the names of CDC2, CDC28A, cell division cycle 2 homolog A, p34 protein kinase, and p34—can form a complex with cyclins A, B, D, and E [13]. The kinase regulates G1/S and G2/M phase transition and promotes M phase progression [24]. It is regarded as the master regulator of the cell cycle, because its functions cannot be compensated by other CDKs, including the closely related CDK2 [25]. In contrast, CDK1 can compensate for the functions of other CDKs, and has been shown to drive mammalian cell cycle progression in the absence of other CDKs [26]. Although the main function of CDK1 is to regulate entry into and progression through mitosis, this protein can also form a complex with other cyclins, such as, D1, E, and A, to regulate G1 phase progression and G1/S transition [23]. During the late G2 phase of the cell cycle, increased levels of cyclin B allow stable CDK1/cyclin B complex formation [20]. This complex is kept inactive by Wee1- and Myt1-dependent inhibitory phosphorylation of the tyrosine 14 and 15 residues in the CDK1 subunit, which interfere with ATP alignment [23,27]. When CDC25 removes the inhibitory phosphates, the complex becomes activated, which induces G2/M phase transition. To exit the M phase, CDK1/cyclin B complex activity needs to be downregulated again, which is achieved through cyclin B proteolysis. The activated CDK1/cyclin B complex is capable of phosphorylating over 100 different proteins [20]. In addition to cell cycle-related targets, the complex has been shown to phosphorylate proteins involved in cell migration and cytoskeleton regulation [28]. Furthermore, CDK1 is emerging as a key regulator of self-renewal and differentiation of human embryonic stem cells (hESCs) and human induced pluripotent stem cells (hiPSCs) [25,29]. Although the knowledge on CDK1 has increased considerably over the last decade, not all processes in which CDK1 is involved in are already understood. CDK1 overexpression likely causes PDAC tumorigenesis by stimulation of checkpoint evasion and induction of stemness properties.

### 2.2. Bypassing the Cell Cycle Checkpoint

**Figure 2:**
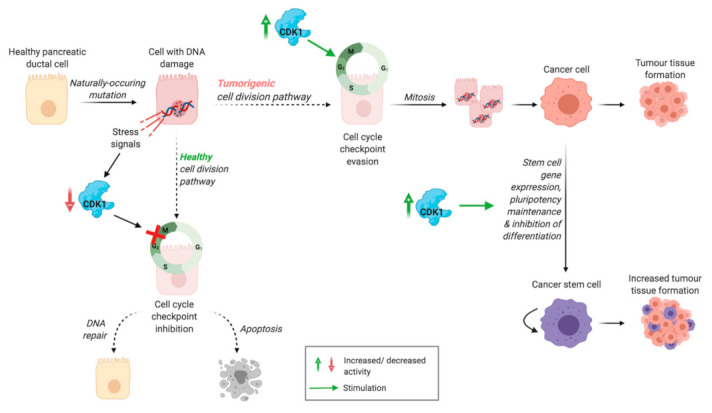
Proposed tumorigenic activity of CDK1. Healthy cell division pathway: stress signals induced by DNA damage lead to inactivation of CDK, inhibiting the cell cycle checkpoint. This results in DNA repair or apoptosis, preventing replication of cells with DNA damage. Tumorigenic cell division pathway: CDK1 overexpression induces cell cycle checkpoint evasion, leading to proliferation of cells with DNA damage, which can eventually lead to tumour tissue formation. Pluripotency maintenance and stem cell gene expression, stimulated by CDK1 activity, contribute to the development of cancer stem cells, increasing tumour tissue formation.

To ensure genomic integrity, several checkpoints regulate cell cycle progression, including the DNA structure checkpoint, also called the G2/M phase checkpoint [27]. Under normal conditions, CDK1 binds to cyclin B to mediate progression from the G2 to M phase of the cell cycle [12,13]. In a situation of cell stress or DNA damage, the activation of the CDK1/cyclin B complex is delayed by Chk1 and Chk2, which inhibit CDC25 and upregulate Wee1 and Myt1 [27]. This induces cell cycle arrest at the G2/M phase and DNA repair, after which the cell cycle can continue. In case of impaired DNA repair, the cell undergoes apoptosis to ensure that a cell with faulty DNA is not replicated. However, CDK1 overactivation might allow evasion of this checkpoint, even with DNA damage [30,31]. In a healthy cell, both of these pathways, regulated by (lack of) CDK1 activity, ensure that a cell with faulty DNA is not replicated (Figure 2). Since CDK1 activity promotes cell cycle progression through the G2/M cell cycle checkpoint, it is hypothesised that enhanced CDK1 activity allows cells with DNA damage to progress through this checkpoint, which can potentially be tumorigenic (Figure 2) [13]. In a physiological situation, multiple CDKs have the function of regulating progression through the different cell cycle checkpoints. However, in absence of other CDKs, CDK1 alone is sufficient to drive mammalian cell cycle progression [26]. This suggests that overexpression of CDK1 on its own is sufficient to drive cell cycle progression of cancer cells, without the need for other CDKs to be overexpressed. 

### 2.3. Inducing G2/M Phase Cell Cycle Arrest

The first proposed anticancer mechanism of CDK1 inhibition is halting cell cycle progression of tumour cells at the DNA structure checkpoint, resulting in G2/M phase cell cycle arrest. Various studies described the effect of CDK1/cyclin B complex activity inhibition on cancer cell cycle proliferation. 

Two compounds, RO-3306 and BA-j, specifically inhibit CDK1 without significantly affecting the activity of other CDKs [20,32]. The effect of these agents on PDAC cell lines was never investigated. They showed promising effects on other tumour cell lines, through induction of G2/M phase cell cycle arrest mediated by RO-3306 [20,33]. The structurally modified flavonoid BA-j inhibits CDK1 activity directly and indirectly, through inactivation of CDC25 [32]. Direct inhibition of CDK1 activity in PDAC cell lines has only been tested with CDK1 small interfering RNA (siRNA), which decreased survival of PDAC cell lines with a KRAS mutated form [34].

Drugs which inhibit multiple CDKs have been tested more extensively. Huang [18] reported that dinaciclib, a CDK 1/2/5/9 inhibitor, induced G2/M phase cell cycle arrest in human PDAC cell lines. This evidence was confirmed by the study of Parry et al. [16] which investigated the effect of dinaciclib on tumour lines of diverse origins, including pancreatic cancer, reported that dinaciclib treatment induced cell cycle arrest, with a relatively high percentage of cells in G2/M phase, at the expense of S phase. In contrast, Khan et al. [19] found that treatment of human PDAC cell lines with dinaciclib generally resulted in an increased proportion of cells in the S phase, which is associated with the blockage of CDK2 activity. Since dinaciclib inhibits multiple CDKs, these results may suggest that the effect of the drug is dependent on the mutations present in the PDAC cell type used. This explanation is further confirmed by the observation variable results that were found for the different PDAC cell lines. In the Capan-1 cell line, the dinaciclib treatment did not induce S phase cell cycle arrest, and in the FA-6 cell line, the treatment did induce G2/M phase cell cycle arrest. The proportion of cells in the sub G1 phase was elevated in all cell lines tested, indicating that many cells were in an apoptotic state. Despite the different effect of dinaciclib in the PDAC cell lines, it is possible to say this drug significantly inhibited proliferation of PDAC cells and the drug was identified as a potent cytotoxic agent against PDAC [16,18,19]. 

Aside from direct targeting of CDK1 activity, there are multiple drugs for which the anticancer effect is proposed to be mediated at least partially through inhibition of CDK1. These drugs include 5MeOlndox, HDAC inhibitors, and flavonoids. 5MeOlndox has been shown to induce G2/M phase cell cycle arrest and reduce proliferation in both mouse and human PDAC cell lines [35]. The arrest of cells in G2/M phase was accompanied by a reduced level of CDK1 and cyclin B1, suggesting that the mechanism of tumour growth inhibition was mediated through reduced CDK1/cyclin B1 activity. Another study, examining the potential of treatment with silibinin and the HDAC inhibitor TSA, found that this combination effectively inhibited cell growth in human pancreatic cancer cell lines [36]. The treatment reduced the expression of cyclin A1, cyclin B1, and CDK1, and induced G2/M phase cell cycle arrest. This reduced expression of CDK1 and cyclin B1 suggests that the lack of activity of these proteins caused the cells to be arrested at the DNA structure checkpoint, resulting in G2/M phase cell cycle arrest. However, the expression of other CDKs and cyclins was not measured, which means it is not possible to assess the effect of (lack of) activity of other CDKs and cyclins. 

### 2.4. Inducing Apoptosis

The existing literature on the effect of CDK1 inhibition on apoptosis reports some controversies. Under healthy conditions, it is assumed that CDK1 is required for the proper execution of apoptosis in the case of DNA damage or a condition of cell stress [13]. In cancer cells, the role of CDK1 in the apoptotic pathway is more complicated. CDK1 can phosphorylate both pro- and anti-apoptotic proteins, and the effect of this phosphorylation can be either activating or inhibitory [31,37]. Simultaneously, CDK1 can regulate transcription of proteins involved in apoptotic pathways, influencing the expression of these proteins. 

**Figure 3:**
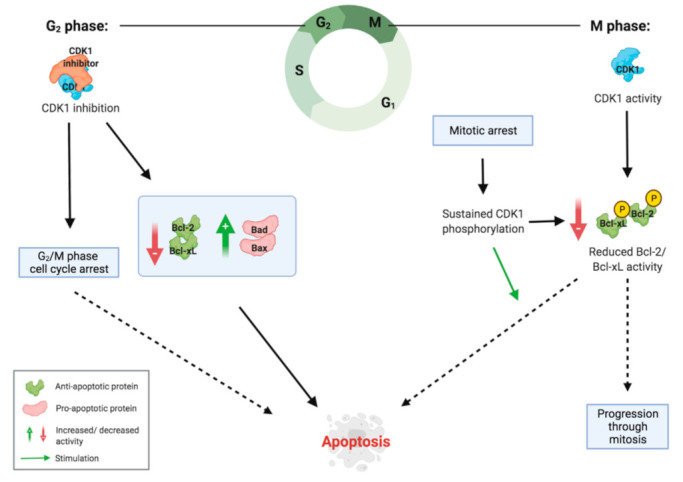
Role of CDK1 in apoptosis induction during the G2 and M phase. During the G2 phase, CDK1 inhibition leads to a decrease in anti-apoptotic proteins and an increase in pro-apoptotic proteins, stimulating the intrinsic apoptotic pathway. Furthermore, lack of CDK1 activity induces G2/M phase cell cycle arrest, which can also lead to apoptosis. During the M phase, CDK1 activity phosphorylates anti-apoptotic proteins in low amounts, inactivating them. In the situation of mitotic arrest, this phosphorylation is sustained, reaching high enough levels of inactivated anti-apoptotic proteins to induce apoptosis.

There is substantial evidence that CDK1 activity phosphorylates the anti-apoptotic proteins Bcl-2 and Bcl-xL during mitosis [31,38,39]. When this phosphorylation is sustained, for example during mitotic arrest, this leads to inactivation of Bcl-2 and Bcl-xL and subsequently induces apoptosis [38]. This explains how, if cells are arrested in mitosis, CDK1 activity can switch mitotic arrest to apoptosis (Figure 3). This crucial role of CDK1 in inducing apoptosis helps to explain several studies reporting an association between absence of CDK1 expression in tumour tissue and decreased survival rates of patients.

In contrast to this, there are also studies that indicate how the lack of CDK1 activity can stimulate apoptosis. Parry et al. [16] found that treating pancreatic cancer cells with dinaciclib resulted in detectable caspase activation in 11 of 15 cell lines tested. Similarly, treating human PDAC cell lines with dinaciclib was shown to induce caspase 3 activation, which is part of the intrinsic pathway of apoptosis [18]. In both studies, the treatment was also associated with G2/M phase cell cycle arrest, suggesting that the induction of apoptosis was mediated via the DNA structure checkpoint. Another study showed that treatment of human pancreatic cancer cell lines with the HDAC inhibitor TSA and the flavonoid silibinin led to a downregulation of Bcl-xL, which was accompanied by inhibition of CDK1 [36]. These results combined indicate that in the situation of G2/M phase cell cycle arrest, a lack of CDK1 activity contributes to the initiation of apoptotic pathways, whereas during mitotic arrest, CDK1 activity is necessary for inducing apoptosis (Figure 3).

### 2.5. Inducing and Maintaining Cancer Stem Cell Properties

CDK1 has been shown to play an important role in the maintenance of pluripotency in human pluripotent stem cells [25,40]. Next to this, high levels of CDK1 are associated with the pluripotency stage of embryonic stem cells [29]. Pluripotency and self-renewal are important properties of cancer stem cells (CSCs), which play a crucial role in the development and metastasis of PDAC [4]. The elevated ability of pancreatic cancer stem cells to self-renew contributes to enhanced tumour growth, which leads a to worse prognosis for patients (Figure 2). Moreover, the capability of CSCs to differentiate into heterogenous cancer cells contributes to tumour heterogeneity, which makes tumours more resistant to treatment [41]. 

**Figure 4:**
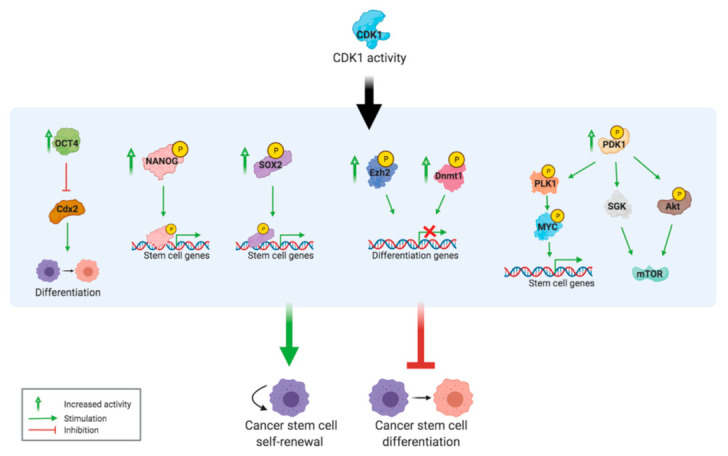
CDK1 overexpression induces cancer stem cell properties. The different mechanisms through which CDK1 overexpression contributes to the formation and maintenance of CSCs.

There are several mechanisms through which CDK1 has the ability to induce pluripotency and inhibit differentiation in PDAC cells (Figure 4). Firstly, CDK1 is thought to directly influence the activity of pluripotency factors (Oct4, NANOG, and Sox2), while inhibiting differentiating factors (Cdx2) [42]. The CDK1/cyclin A complex can directly phosphorylate NANOG, promoting activity of the protein. Menon et al. [43] observed that CDK1 activity increased Sox2 phosphorylation and nuclear translocation, which induced the transcription of stem cell genes. Moreover, Sox2 is related to tumour initiation, therefore an excessive expression of CDK1 promotes the development of CSCs in the tumour microenvironment by Sox2 activity [43]. Multiple studies have shown that upon differentiation of embryonic stem cells, CDK1 mRNA and protein levels decreased following the same pattern as the pluripotency factors Oct4, NANOG, and Sox2 [25,29,43]. This further confirms the link between CDK1 activity, these pluripotency factors, and pluripotency maintenance. 

Moreover, CDK1 has the potential to influence epigenetic regulation via phosphorylation of DNA methyltransferase 1 (Dnmt1) and Enhancer of zeste homolog 2 (Ezh2), thus to contribute to the transcriptional induction of pluripotency [12].

Wang et al. [29] reported that 3′-phosphoinositide-dependent protein kinase-1 (PDK1) is a substrate of CDK1 and that CDK1 activity stimulates the PDK1/Akt/mTOR signalling pathway for self-renewal. Importantly, they noted that this process is unrelated to the effect of CDK1 on the cell cycle, because at a reduced level of CDK1 where the cell cycle was unaffected, pluripotency was already reduced. PDK1 also phosphorylates other targets, including the activation site of Akt [44]. Akt regulates many cellular processes like cell growth, cell proliferation, and survival through its effect on mTOR, and this pathway often acts as a drug and radiation resistance mechanism of cancer cells when the Akt signalling pathway is impaired [45]. Another tumorigenic effect of PDK1 is mediated through activity of the MYC protein, which is overexpressed in many human cancers and is considered a central oncogene in PDAC [46,47]. MYC has also been identified as one of the main driving forces behind the development of pancreatic CSCs, which show high metastatic potential [48]. PDK1 activity induces phosphorylation of PLK1, which phosphorylates and stabilizes the MYC protein. This leads to oncogenic transformation, partially through upregulation of cancer stem cell-like genes.

### 2.6. Targeting Cancer Stem Cells 

Another mechanism through which CDK1 inhibition is proposed to work is by suppressing the effects of pluripotency maintenance induced by an enhanced CDK1 activity. Differentiation of cancer stem cells and loss of pluripotency could transform CSC into regular cancer cells, which may not replicate infinitely. 

Menon et al. [43] showed that for melanoma cells, inhibition of CDK1 via AZD5438 decreased the tumour-initiating capacity, which is also a key characteristic of pancreatic CSCs [49]. This decrease in tumour-initiating capacity was mediated through reduced activation of Sox2, a key regulator of stem cell gene transcription. Casari et al. [50] showed that PDK1 inhibition impairs cell proliferation and colony formation of pancreatic cancer cell lines. Since CDK1 stimulates PDK1 activation, this indicated that CDK1 inhibition will impair cell proliferation and colony formation via reduced PDK1 activity, which induces differentiation of CSCs. This was further confirmed by another study, which showed that the silencing of CDK1 reduced tumour growth of hepatocellular carcinoma CSCs by inhibiting their clonogenic potential and self-renewal ability [41]. This anticancer effect was mediated through a downregulation of PDK1, β-Catenin, and Akt. Moreover, they established an interaction between both CDK1 and PDK1 and PDK1 and Akt. 

Examination of the effects of CDK1 inhibition on embryonic stem cells (ESCs) can contribute to understanding the impact of this on CSCs. The effect of CDK1 inhibition on ESCs has been studied using both siRNAs and small molecule inhibitors. CDK1 inhibition results in an increased percentage of ESCs in the G2/M phase [25,51]. Neganova et al. [25] demonstrated that inducing CDK1 siRNA resulted in a loss of pluripotency in human ESCs. The loss of pluripotency was accompanied by a reduction in Oct4 and an increase in Cdx2. This suggests that CDK1 inhibition caused elevated Cdx2 activity via Oct4 downregulation, inducing differentiation. In contrast, Huskey, et al. [51] did not find evidence that CDK1 siRNAs promoted differentiation of mouse ESCs. This was explained by another study, showing that elongation of the ESC cell cycle does not induce differentiation [52], explaining that induction of G2/M phase cell cycle arrest via CDK1 inhibition does not have an effect on the pluripotency of ESCs. However, when CDK1 overexpression contributes to pluripotency maintenance of CSCs, inhibition of CDK1 can potentially still induce differentiation. 

Thus, there is limited research suggesting that CDK1 inhibition induces differentiation of pancreatic cancer stem cells. However, the literature suggests that CSCs have increased sensitivity towards CDK1 inhibition compared to non-stem cells. This is promising for the application of CDK1 inhibitors to treat PDAC, because efficient and specific targeting of pancreatic CSCs has high therapeutic potential [49]. 

## 3. Therapeutic Potential

### 3.1. CDK1 Inhibitors 

Since dysregulation and overexpression of protein kinases play an essential role in the prognosis of many types of cancer, including PDAC, in recent years many kinases inhibitor small molecules have been synthesized and advanced into clinical trials.

To date, the food and drug administration (FDA) has approved 62 small molecules protein kinase inhibitors. Among the 62 approved drugs, 35 small molecules are receptor protein-tyrosine kinase inhibitors, 13 small molecules target non receptor protein-kinase inhibitors, 10 small molecules are serine/threonine kinase antagonists, and 4 are dual protein kinase inhibitors (MEK1/2) [53].

During the last years, great progress has been made also in developing CDK inhibitors; however, among CDKs, the only FDA approved drugs work by inhibiting CDK4/CDK6 (abemaciclib, ribociclib, and palbociclib) and no CDK1 inhibitor has reached the market [53]. Moreover, the high degree of similarity shared between the ATP binding site of CDKs represented a challenge to generating selective compounds. Therefore, the first generation of CDK inhibitors developed showed activity across multiple CDKs and were defined as Pan-CDK inhibitors [54].

Flavopiridol, also known as Alvocidib, is a synthetic flavonoid based on the chromone alkaloid, rohitukine. It was one of the first CDKs inhibitors developed that exhibited potent inhibition of CDK 1, 2, 4, 6, 7, and 9, with IC_50_ values of 30, 20, 60, 10, 10 nM, respectively.

**Figure 5:**
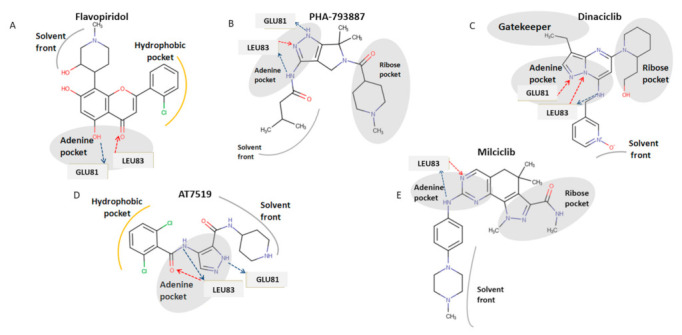
Structures of CDKs inhibitors and proposed binding mode of these compounds binding with CDK1. (**A**) Chemical structure of flavopiridol and its depicted binding mode with CDK1. (**B**) Chemical structure of PHA-793887 and its depicted binding mode with CDK1. (**C**) Chemical structure of Dinaciclib and its depicted binding mode with CDK1. (**D**) Chemical structure of AT7519 and its depicted binding mode with CDK1 (**E**) Chemical structure of milciclib and its depicted binding mode with CDK1. Hydrogen bonds donor interactions are indicated by blue arrows, hydrogen bonds acceptor interactions are indicated by red arrows and amino acid residues that interact with inhibitors through hydrogen bonds are shown in grey backbone.

The X-ray crystal structure of CDK2 in complex with flavopiridol revealed the molecular structure features for the inhibition. In particular, the oxygen O4 and hydroxyl group at 5 position of flavones moiety bind with the hinge residues of leucine (Leu) 83 and glutamate (Glu) 81, whereas the piperidinyl group is exposed to the solvent region [55] (Figure 5A).

PHA-793887 is a pyrrolopyrazole derivative that mainly inhibits CDK2-cyclinA, CDK2-cyclinE, CDK5-p25, and CDK7-cyclinA, with IC_50_ values of 80, 80, 5, 10 nM, respectively. Moreover, PHA-793887 is able to inhibit CDK1-cyclin B, CDK4-cyclinD1, CDK9/cyclin T1, and GSK3-β with IC_50_ values of 60, 62, 138, and 79 nM. Regarding the binding mode of PHA-793887 with CDK1, the pyrazole moiety occupies the adenine region of the ATP binding site. In particular, the amino group of burinamide forms a hydrogen bond with the backbone of Leu 83, while the nitrogen and amino group of the pyrazole makes two additional hydrogen bonds with the carbonyl oxygen of Glu 81 and with the amino group of Leu 83 at the hinge region, while the isobutyl group points toward the solvent accessible region [56] (Figure 5B).

Dinaciclib is a pyrazolopyrimidine derived inhibitor of CDK 1, 2, 5, and 9, with IC_50_ values of 3, 1, 1, 4 µM, respectively [16]. Dinaciclib is a type I inhibitor binding the ATP site through the pyrazolopyrimidine moiety that makes a hydrogen bond with Leu 81–83 of the hinge region. The 3-ethyl group of the pyrazolopyrimidine moiety establishes hydrophobic interactions with the gatekeeper residues of Phe 80. The pyridine oxide ring is exposed to the solvent region [57] (Figure 5C). 

AT7519 was discovered through fragment-based screening approaches by Astex as CDK2 inhibitor with IC_50_ values of 47 nM, further studies demonstrated that this drug is also a potent on CDK 1, 4, 6, and 9 with IC_50_ values of 210, 100, 13, and 170 nM, respectively. The binding mode of AT7519 with CDK1 is that of a classic competitive inhibitor, with the carbonyl of 4-benzamide group that makes two hydrogen bonds with Leu 83 donor-acceptor. A further interaction occurs between the amino group of pyrazole ring and Glu 81 of the hinge region [58,59] (Figure 5D).

Milciclib (PHA-848125) is a multi-CDK inhibitor with IC_50_ values of 45, 160, 265, 363, 398, and 150 nM against CDK2-cyclinA, CDK4-cyclinD1, CDK5-p35, CDK2-cyclinE, CDK1-cyclinB, and CDK7-cyclinH, respectively. The proposed binding mode of this drug was confirmed by X-ray crystal structure of CDK1 in complex Milciclib. The pyrazoloquinazoline moiety of the molecule occupies the ATP binding site and makes hydrogen bonds with the backbone NH of Leu83, while the adjacent amino group binds to the carbonyl oxygen of Leu83 (Figure 5E) [60].

### 3.2. Preclinical Studies on CDK1 Inhibition

Multiple studies mentioned in the previous paragraphs demonstrated the efficacy of drugs targeting CDK1 inhibition on reducing PDAC cell growth in vitro [16,18,19,35,36]. In vivo mice experiments have also elicited promising results for CDK1-targeting drugs. Administration of dinaciclib, an inhibitor of CDKs 1/2/5/9, inhibited tumour growth in 10 out of 10 subcutaneous PDAC mouse models tested, with significant growth reduction (>40%) in 8 out of 10 [61]. Similarly, dinaciclib treatment delayed tumour progression and increased the overall survival from 31 to 57 days in a transgenic mouse model of PDAC [62]. Moreover, treatment induced apoptosis of tumour cells in vivo, next to inhibition of cell proliferation. Huang et al. [18] found that dinaciclib treatment in combination with immunotherapy improved survival rate and reduced tumour formation in subcutaneous, orthotopic, and transgenic PDAC mouse models. The treatment induced more effective tumour reduction than paclitaxel, a drug (as nab-paclitaxel) also used to treat PDAC patients. At half of the maximum tolerated dose for both drugs, paclitaxel showed a tumour growth inhibition of only 63 percent, compared to 90 percent for dinaciclib. Furthermore, dinaciclib prevented tumour formation and decreased the growth of established stem-cell derived tumours in mice [51]. The treatment selectively killed stem cell-derived tumour components, which shows potential for targeting CSCs via CDK1 inhibition. Together, these studies illustrate that the compound dinaciclib has the ability to significantly reduce PDAC cell growth in vivo.

Sano et al. [35] tested two other CDK1-inhibiting drugs, Indox and 5MeOIndox, on mice with subcutaneously transplanted PDAC cells. Both drugs inhibited tumour growth and reduced tumour weight, but only 5MeOIndox reduced CDK1/cyclin B1 complex levels. The authors concluded that treatment with 5MeOIndox was more promising to treat PDAC, because it induced early apoptosis as opposed to late apoptosis of Indox. Wu et al. [41] reported that in combination with sorafenib, RO 3306 CDK1 inhibitor decreased the population of CSCs both in vivo and in vitro, which was accompanied by a reduction in the stemness-related proteins Oct4, Sox2, and NANOG. Administration of the CDK 1/2/4/5 inhibitor purvalanol A was shown to specifically target Oct4 and NANOG-expressing cells and reduce tumour incidence in a population of subcutaneous teratoma xenograft mice [51]. Both studies suggest that CDK1 inhibition can effectively target CSCs in vivo. 

When evaluating whether CDK1 suppression is a propitious drug target, it is crucial to assess the effect of CDK1 inhibition on healthy cells. Prevo et al. [20] investigated the effects of RO-3306 on both healthy and tumour cells. They found that RO-3306 can affect the survival of healthy cells, but only when they are proliferating. This implies a narrow therapeutic window. However, after a long 72 h of exposure to RO-3306, 40% of the cancer cells were in an apoptotic state but only 10 percent of normal cells, suggesting some selectivity of CDK1 [20]. Conversely, Sano et al. [35] found that 72 h of exposure to 5MeOIndox of both mouse and human PDAC cells led to a significantly impaired proliferation, while healthy mouse fibroblasts were unaffected. Moreover, in vivo, no significant weight loss was observed in any of the treated mice, suggesting a good tolerability of the drug. Additionally, in some other studies CDK1 inhibition was well-tolerated in vivo. For instance, BA-j caused no significant side effects when administered at a therapeutic dose range [32]. Similarly, dinaciclib was well tolerated in multiple in vivo studies [16,63,64]. Obviously, toxicity of CDK1 inhibition needs to be carefully investigated in additional preclinical trials before moving to the clinic.

### 3.3. Clinical Trials of CDK1 Inhibitors

Thus far, the promising results of the preclinical studies with CDK1 inhibitors have not yet been translated properly to the clinic as a novel potential treatment for PDAC. Table 1 gives an overview of completed clinical trials testing the efficacy and tolerability of different non-specific CDK1-inhibitors, since all drugs inhibit multiple CDKs. The most widely-tested drug is dinaciclib, a small molecule inhibitor of CDKs 1/2/5/9. This drug showed promising efficacy and good tolerability in phase II clinical trials for myeloma and phase III clinical trials for chronic lymphocytic leukaemia [65,66]. However, even though generally well-tolerated, dinaciclib was less effective in solid tumours, including pancreatic cancers [67,68,69]. In a combination treatment of dinaciclib with the Akt-inhibitor MK-2206, the best clinical result was stable disease in only four pancreatic cancer patients (10 percent) with a median survival rate of 2,2 months [68]. Similar poor results have been found for flavopiridol, an inhibitor of CDKs 1/2/4/6, in a phase II clinical trial on pancreatic cancer [70], since the combination treatment of flavopiridol and docetaxel generated no objective responses, and only three patients (33 percent) achieved stable disease, with a median survival rate of 4,2 months. Both the CDK 1/2/9 inhibitor AZD5438 and the CDK 1/2/4 inhibitor PHA-793887 showed no clinical benefit in phase I studies with patients with solid tumours [71,72], since in both clinical trials serious adverse effects were observed, leading to deaths. 

However, the CDK1/2/4/5 inhibitor milciclib in combination with gemcitabine showed some clinical benefit in a phase I study on patients with refractory solid tumours [73]. Of the 16 patients, 43 percent showed stable disease, including long-term disease stabilisation for a pancreatic cancer patient of over 6 months. The combination treatment was well-tolerated with manageable toxicities. Comparable results have been found in a phase II study with hepatocellular carcinoma patients [74]. Furthermore, the CDK 1/2/4/5/7 inhibitor PHA-848125AC showed some efficacy (two partial responses in patients with thymic carcinoma) in a phase I clinical trial with patients with advanced solid tumours [75]. Interestingly, the pancreatic cancer patients showed, on average, stable disease for 10 months. 

Hence, even though the preclinically promising drug dinaciclib has failed to show sufficient anticancer activity in clinical trials for PDAC, there is evidence suggesting that other CDK1-inhibiting drugs can contribute to improved treatment of this disease. 

### 3.4. Importance of Screening Patients

The limited efficacy of the CDK inhibitors in clinical studies may be related to the lack of selection of patients. For any therapy, but especially for a therapy targeted against CDKs, it seems essential to screen patients in order to ensure that PDAC tumour cells are indeed overexpressing CDK1. This can be done in solid tumour tissue with immunohistochemistry staining for CDK1 [24,68]. Thus, CDK1 overexpression can be used as a biomarker to distinguish between different types of PDAC. Furthermore, siRNA-based screening might help to identify CDK1 as a target in tumour tissue [34,76], but this technique needs to be optimised further before being used to select PDAC patients likely to respond to CDK inhibition. In many clinical trials, patients are not screened for CDK1 overexpression [68,69,73]. Although Mita et al. [67] demonstrated that it is feasible to include skin and tumour biopsy immunohistochemistry staining in a clinical trial, they did not apply analysis of CDK1 expression in their study. In order to develop effective therapies targeting CDK1 inhibition, clinical trials should measure the CDK1 tumour expression of PDAC patients before and during treatment in order to keep track of how CDK1 expression is affected by the therapy.

### 3.5. Combination Therapy 

**Figure 6:**
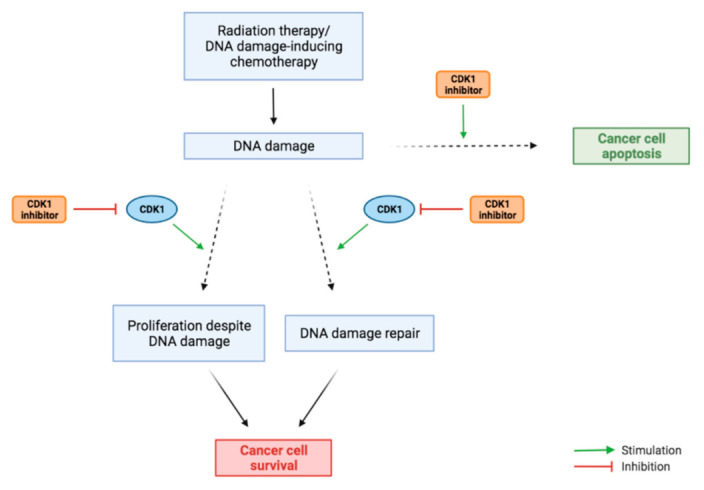
Interaction of CDK1 inhibitors in combination with radiation treatment and chemotherapy. CDK1 overactivity stimulates DNA damage repair and the proliferation of cells despite DNA damage (via cell cycle checkpoint evasion), leading to survival of cancer cells. CDK1 inhibitors combat this effect, stimulating the pathway from DNA damage which leads to apoptosis.

Potentially, CDK1-targeted drugs can be combined with conventional chemotherapy, other forms of targeted therapy, or ionising radiation therapy. CDK1 inhibition can sensitise tumour cells to radiation [20]. Neganova et al. [25] showed increased apoptosis of human ESCs treated with a CDK1 inhibitor only when the drug was combined with radiation, which induced DNA damage. This might be related to the important role of CDK1 in DNA damage repair. The combination of inducing DNA damage through radiation and inhibition of the DNA damage repair through CDK1 inhibition could potentially be used to increase apoptosis in PDAC cells (Figure 6). Furthermore, cells are more susceptible to DNA damage induced by radiation during G2/M phase cell cycle arrest, which is a result of CDK1 inhibition [77]. Additionally, there is evidence that CDK1 overexpression can induce resistance to radiation therapy, which is reversed by knocking out CDK1 [78]. Taken together, these studies imply that CDK1 inhibitors can sensitise tumour cells for radiation therapy, increasing the efficacy of this therapy. 

Regarding several forms of chemotherapy, the combination of existing treatments with CDK1-inhibiting drugs may have multiple potential benefits. Firstly, CDK inhibitors can prevent recovery of cells after DNA damage-inducing chemotherapy, enhancing the efficacy of the treatment [4], possibly via the same mechanism of inhibition of the DNA damage repair pathway as described for radiation therapy [77]. Specifically, CDK1 inhibition can sensitise cells to treatment with aphidicolin and cisplatin [79]. Secondly, CDK1 inhibition can sensitize specific cancer types that would be unsensitive by a specific form of chemotherapy alone, extending the therapeutic spectrum of these drugs. The PARP inhibitor olaparib, which is considered only effective against BRCA-mutated cells, showed enhanced cytotoxicity in BRCA-proficient cells in combination with RO-3306 [79]. Mayes et al. [80] showed that CDK1 sensitises cancer cells to TRAIL-induced apoptosis. These effects are mediated by the crucial role of CDK1 in survival of proliferating cells. The PARP inhibitor-induced DNA damage does not lead to apoptosis when enhanced CDK1 activity stimulates cell cycle progression, regardless of DNA damage. However, when this cell cycle checkpoint evasion is blocked by CDK1 inhibitors, the cancer cells are less likely to survive (Figure 6). This also explains studies demonstrating that CDK1 inhibition reduces gemcitabine- and 5-FU resistance in multiple different cancer types [73,80,81,82]. Another study showed that CDK1 inhibition enhanced the ability of the protein kinase inhibitor sorafenib to specifically kill stem cells of hepatocellular carcinoma [41]. This highlights the potential of this combination treatment to eliminate cancer stem cells in malignant solid tumours, which is beneficial for treating PDAC. 

CDK1 inhibition will not work in combination with every treatment. For example, when combined with drugs that induce mitotic arrest, such as taxanes, CDK1 inhibition might have an adverse effect, due to its role in apoptosis initiation during mitosis. This illustrates the importance of carefully selecting a combination of therapies and focusing on the biochemical effects of their interaction.

## 4. Concluding Remarks

PDAC has a poor survival rate due to the lack of effective and tolerable treatment. Therefore, there is an urgent need to develop new therapies for this disease [4,13]. The overexpression of CDK1 genes suggests a role for this cyclin-dependent kinase in PDAC development and growth [14]. Hence, this literature review evaluated the potential of CDK1 inhibition for novel drug development to treat PDAC. 

CDK1 overexpression exerts its tumorigenic effect predominantly via two mechanisms. Firstly, through stimulation of cell cycle progression at the G2/M phase checkpoint, leading to cell proliferation of cells with a potential tumorigenic mutation. Secondly, by inducing pluripotent characteristics in PDAC cells, leading to the development of CSCs. These CSCs promote tumour initiation, tumour growth, and heterogeneity of tumours, making them more challenging to treat [4,41]. Multiple studies showed the ability of CDK1 inhibitors to induce G2/M phase cell cycle arrest and apoptosis in tumour cells, both in vitro and in vivo. This effect was shown for many different cancer types, including PDAC. Furthermore, multiple studies suggest that CSCs might be especially sensitive toward CDK1-inhibiting drugs. The specificity of CDK1 inhibitors for pluripotent cells is beneficial when killing CSCs, but potentially very dangerous for healthy stem cells. Next to this, there are concerns about the potential toxic effect of CDK1 inhibition on healthy cells in general [20]. Thus, more research on the effects of CDK1 inhibition on healthy human (pluripotent) cells is needed. 

An assessment of clinical trials revealed that CDK1 inhibitors failed to show sufficient response in patients with PDAC. The lack of efficacy of CDK1 inhibitors despite the strong preclinical data to support their use may be related to the poor pharmacokinetics of the drugs [67,69]. Another explanation for the lack of efficacy could be the rapidly progressive nature of PDAC. Additionally, clinical trials are usually performed on PDAC patients that failed to respond to other treatments. This often means that they are in an advanced stage of tumour progression and these patients might also be more resistant to therapy in general, reducing the chances that the treatment will be effective. To increase the potential of CDK1 inhibitors to reduce PDAC tumour growth, screening of patients for CDK1 overexpression is important for future clinical trials. 

CDK1 inhibition might be best used in combination with other therapies. Promising results have been found for the combination of CDK1 inhibition with both ionising radiation therapy and DNA damaging chemotherapy. Reduction of CDK1 activity can both sensitise cancer cells to treatment and enlarge the therapeutic spectrum of existing therapies. This is promising for the use of CDK1 inhibition to overcome drug resistance, which is regarded as one of the main causes for the poor prognosis of PDAC patients [83]. However, inhibition of CDK1 will not work in combination with every treatment, and might even have adverse effects, for example when combined with drugs that induce mitotic arrest. The knowledge on molecular mechanisms of CDK1 inhibition can contribute to improve the selection of proper treatment combinations for different types of PDAC that needs further characterisation. This knowledge will also help to establish a lower minimal effective dose, which can limit the cytotoxic effects of the drugs on healthy cells. 

In conclusion, CDK1 inhibition seems to be a propitious drug target for the treatment of PDAC. The anticancer mechanism is mediated through the induction of G2/M phase cell cycle arrest, induction of apoptosis, and specific targeting of cancer stem cells. At the moment, there are multiple clinical trials ongoing investigating the efficacy and tolerability of CDK1-inhibiting drugs, alone and in combination with other therapies. Next to this, screening possibilities for CDK1 overexpression need to be implemented to improve a personalized medicine approach that is essential to effectively apply these drugs.

## Figures and Tables

**Table 1:** Clinical trials testing different CDK1-inhibiting drugs.

Drug (Targeted CDKs)	Clinical Trial Phase	Disease (Number of Patients)	Efficacy	Tolerability	Observations	Reference
AZD5438 (1, 2, 9)	Phase I	Advanced solid tumours (64, 8 pancreatic)	−	− −		[71]
Dinaciclib (1, 2, 5, 9)	Phase I	Pancreatic cancer (39)	−	+	In combination with MK-2206 (Akt inhibitor)	[68]
		Advanced malignancies (61, 5 pancreatic)	− +	+		[67]
		Advanced malignancies(48, 4 pancreatic)	− +	− +		[69]
	Phase II	Advanced breast cancer (39)	− +	+	In comparison vs. capecitabine (similar but not superior anticancer activity)	[67]
		Refractory multiple myeloma (27)	++	+		[66]
	Phase III	Refractory chronic lymphocytic leukemia (42)	+ +	+	In comparison vs. ofatumumab (results suggest superior anticancer activity)	[65]
Flavopiridol (1, 2, 4, 6)	Phase II	Pancreatic cancer (10)	+ −	− −	In combination with docetaxel	[70]
Milciclib (1, 2, 4, 5)	Phase I	Refractory solid tumours (16, 13 pancreatic)	+	+ +	In combination with gemcitabine	[73]
	Phase II	Hepatocellular carcinoma (14)	+	+ +		[74]
PHA-848125AC (1, 2, 4, 5, 7)	Phase I	Advanced solid malignancies (37, 5 pancreatic)	+	− +		[75]
PHA-793887 (1, 2, 4)	Phase I	Solid tumours (19, 5 pancreatic)	−	− −		[72]

## Data Availability

Data related to the study are included in the article. Data are available from the corresponding authors (E.G. and P.D) upon reasonable request.
